# Characterization of the onset of puberty in Tazegzawt lambs, an endangered Algerian sheep: Body weight, thoracic perimeter, testicular growth, and seminal parameters

**DOI:** 10.14202/vetworld.2018.889-894

**Published:** 2018-07-02

**Authors:** Farid Moulla, Rachid El-Bouyahiaoui, Rebouh Nazih, Norezzine Abdelaziz, Nacera Zerrouki, Mokrane Iguer-Ouada

**Affiliations:** 1 National Institute for Agronomic Research of Algeria (INRAA), 02, Av. Frères Ouaddek, BP, 200, El Harrach, Alger, Algeria; 2 Department of Agronomic, Faculty of Biologic and Agronomic Sciences, University Mouloud Mammeri of Tizi-ouzou, Tizi-ouzou, Algeria; 3 Agrarian and Technological Institute, Peoples Friendship University of Russia, Russia; 4 Laboratoire Associé en Ecosystèmes Marins et Aquacoles, Faculté des Sciences de la Nature et de la Vie, Université de Béjaia, Algérie

**Keywords:** body weight, puberty onset, scrotal circumference, seminal parameters, Tazegzawt breed sheep

## Abstract

**Aim:**

The aim of the present study was to define the onset of puberty in Tazegzawt ram lambs, an Algerian sheep breed in endangered status with a small population in its local area.

**Materials and Methods:**

Body growth (body weight and thoracic perimeter), scrotal circumference (SC), penis development stages, and seminal parameters (volume, concentration, and motility) were measured. Data were recorded at fortnightly intervals in 10 animals from 9 to 49 weeks of age.

**Results:**

On the basis of seminal analyses, puberty occurred between 29 and 45 weeks of age. At 29 weeks of age, 30% of lambs reached puberty, and at 45 weeks of age, puberty was observed in 100% of the analyzed animals. Body weight appeared as the most determinant factor, and the onset of puberty was observed when animals reached 43.2±6.4 kg body weight with 25.8±3.7 cm of SC. Seminal analyses revealed that all parameters increased regularly from puberty onset except for sperm concentration. The mean semen volume during the study period was 0.48±0.33 mL with 0.84±0.6 mL at 37 weeks of age. Sperm concentration evolved similarly as semen volume; at 29 and 43 weeks of age, the sperm concentration was 942×10^6^ and 1904×10^6^ spermatozoa/mL, respectively. Kinematic parameters including the percentage of motility, the percentage of progressive motility, and gametes velocities as determined by Computer-Aided Sperm Analyzer showed the highest values at 49 weeks of age.

**Conclusion:**

The current results revealed that, in Tazegzawt ram lambs, puberty occurs between 29 and 45 weeks when animals reach 43.2±4.6 kg body weight.

## Introduction

The complexity of mechanisms underlying gonads development and sexual maturity in domestic animals reflects the complexity to define accurately onset of puberty particularly in species with seasonal reproduction activity. The interaction between body weight, testis growth [1,2], testosterone secretion [2], and sperm production [3], especially during the pre-pubertal stage, is the key factors influencing puberty. In addition, it has been established that puberty in male lambs depends on genetic origin [4], nutrition [5,6], birth date, and photoperiod length [5]. However, in sheep, puberty seems to be more dependent on body weight considered as the determinant factor [7,8].

Different indicators are commonly used to determine the onset of puberty; nevertheless, the solid yardsticks are the increased testicular size simultaneously to the first presence of spermatozoa in seminiferous tubules, epididymis, and ejaculates [4,9]. Alternatively, puberty is determined on the basis of testicular growth [10], separation of the penis from the prepuce [11], and testosterone concentration [4].

Tazegzawt sheep is an indigenous breed in Algeria well adapted to the local environmental conditions; this breed is in an endangered status with a small population in its origin area located in Kabylia Mountains, in North Center of Algeria. Tazegzawt is a sturdy animal with a large size mainly qualified for meat production. Phenotypically, Tazegzawt breed presents specific characteristics with blue-blackish tasks on the muzzle, the edges of the eyes, and earlobes [12].

To date, no previous characterization of the reproductive physiology of Tazegzawt breed is reported, especially the standards to define accurately puberty onset in ram lambs. Generating knowledge on the reproductive physiology remains a prerequisite to future conservation and promoting strategies, especially through sperm biotechnologies. The aim of the present study was to determinate the puberty onset in Tazegzawt ram lambs on the basis of body growth, gonads index, and sperm output.

## Materials and Methods

### Ethical approval

The experiment was carried out in accordance with the guidelines laid down by the Directive 2010/63/EU of the European Parliament for Animal Ethics Committee for the use of animal experimentation.

### Location

The study was conducted in Béjaïa District, Algeria, at the National Institute of Agronomic Research at an altitude of 26 m above the sea and at a longitude of 36.7070680° E and latitude of 4.958661° N.

### Experimental animals

Ten Tazegzawt ram lambs born in spring (between March and April 2014) from single, twin, or triplet lambing were used. At birth, body weight varied from 4 to 6.30 kg with 5.23±0.72 kg (mean standard deviation [SD]). The animals were used from the age of 9-49 weeks, and ram lambs were weaned at 25 weeks of age.

### Feeding

The ram lambs were maintained with their mothers until weaning and then were housed separately in a shed and raised under the same management and nutritional conditions. The lambs were fed ordinary hay and barley. The water and mineral blocks were available *ad libitum*.

### Data recorded

#### Body weight, thoracic perimeter (TP), and scrotal circumference (SC)

Body weight was measured using small ruminants balance (maximum capacity 200 kg±500 g, Marechalle Pesage, France). TP and SC were measured with a flexible meter tape. The SC was measured around the largest diameter of the scrotum after pushing testes firmly into the scrotum.

#### Evaluation of penis development stages

Three stages were considered to determinate the degree of separation of penile adhesions from the preputial mucosae [9,11]:

Stage 1: Infantile stage: The penis is thin and completely adherent to preputial mucosae.
Stage 2: Separation stage: The penis becomes visible; the separation of adhesions is initiated.
Stage 3: Pre-pubertal stage: The penis is completely separated from the prepuce and is fully exposed through the preputial orifice.

### Electro ejaculation and seminal evaluations

Semen samples were collected at fortnightly intervals by electroejaculation using a battery-powered ram ejaculator (Ruakura probe, length 12 cm, 2.5 cm diameter, Shoof International Ltd., France) delivering a 12 v, 1 amp electric stimulus to the pelvic structures. The stimulation was maintained for 5 s followed by brief equal duration. This sequence was terminated either when an ejaculate was obtained or after 3 to 5 shocks. Electroejaculation was applied from 27 to 49 weeks of age.

The volume of the ejaculate was assessed by a micropipette. Motility and sperm concentration were assessed using a Computer-Aided Sperm Analyzer (Sperm class analyzer, SCA Microptic, S.L., Version 3.2.0, Barcelona, Spain) as follows: The samples were diluted to 20×10^6^ Spermatozoa/mL and 10 µL of each sample was placed in a Makler^®^ chamber (10 µm depth; Sefi Medical Instruments, Haifa, Israel) previously heated (37°C). The chamber was placed under phase contrast microscope (Nikon E200^®^-LED microscope, Spain) and images were captured using a video camera (Basler Digital Camera A312fc Germany) at magnification 10×. Three sequences were scanned, and at least 200 spermatozoa were analyzed. The standard settings were set at 25 frames/s, 20-90 µm^2^ for head area, and curvilinear velocity (VCL) >10 µm/s to classify spermatozoa as motile. Measured kinetic parameters were total sperm motility (TM%), progressive sperm motility (PM%), linearity, straightness (STR%), wobble (WOB%), VCL (µm/s), straight linear velocity (VSL µm/s), average path velocity (VAP µm/s), amplitude of lateral movement of the head (ALH µm), and beat cross frequency (BCF Hertz). TM was defined as the percentage of spermatozoa with VCL >10 µm/s, and PM was defined as the percentage of spermatozoa with VCL >25 µm/s and STR > 80%. Data shown are semen volume, sperm concentration, and VCL velocity.

### Statistical analysis

All statistical analyses were carried out using Statview 4.02 software (Abacus Concepts Inc., Berkeley, CA, USA). The values of body weight, TP, SC, ejaculate volume, sperm concentration, and kinematic parameters were expressed as the mean±standard error of mean. Differences were assessed using one-way ANOVA followed by Fisher’s LSD test. Values were considered statistically significant when p<0.05.

## Results

### Body weight and SC

**Figure-1:**
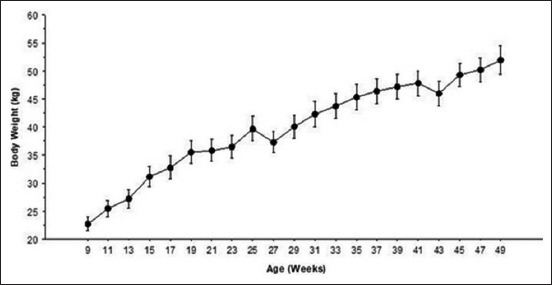
Average body weight of Tazegzawt ram lambs from 9 to 49 weeks of age. Data are presented as mean±standard deviation.

Body weight increased regularly from 9 to 49 weeks of age with 22.8±4.0 kg and 52±2.4 kg, respectively (Figure-1). An average daily gain (ADG) of 104.2±23.1 g/day was recorded when considering the whole study period. During pre-weaning (0-25 weeks) and post-weaning (25-49 weeks), the ADG was 196±37.1 and 37.8±9.2 g/day, respectively.

The growth regression equation curve of body weight according to lambs age was linear with a coefficient of determination R^2^ = 0.61. A slight weight falls were observed between 25 and 27 weeks and between 41 and 43 weeks.

**Figure-2:**
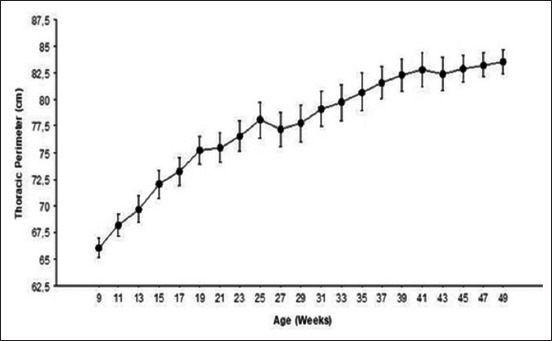
Average thoracic perimeter of Tazegzawt ram lambs from 9 to 49 weeks of age. Data are presented as mean±standard deviation.

TP (Figure-2) showed the same pattern as body weight, and the regression equation according to age revealed a linear relationship with a coefficient of determination R^2^=0.53. From 9 to 49 weeks of age, TP increased from 66±0.9 to 83.5±1.1 cm, with an average daily growth estimated to 0.06±0.09 cm/day.

**Figure-3:**
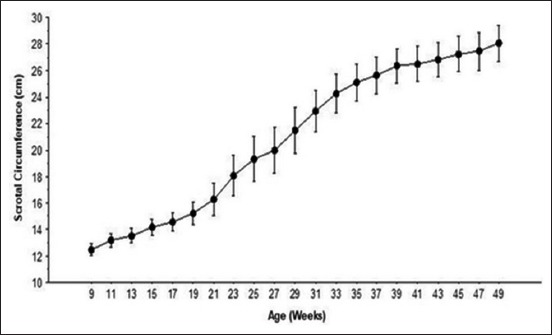
Average scrotal circumference in Tazegzawt ram lambs from 9 to 49 weeks of age. Data are presented as mean±standard deviation.

SC (Figure-3) presented an average daily growth of 0.043±0.091 cm/day; at 9 and 49 weeks of age, values were 12.4±0.43 and 28±1.3 cm, respectively. Three growth rates were observed such as a slight growth from 9 to 21 weeks, exponential growth from 21 to 35 weeks, and again a slight growth from 35 to 49 weeks of age.

### Penis development stages and semen presence

Table-1 represents age, body weight, TP, and SC according to Stages 2 and 3 of penile development. Stage 3 was observed between 27 and 39 weeks in all ram lambs (31.6±5.3 weeks, mean±SD) with 41.2±6 kg, 79±4.4 cm, and 22.6±3.5 cm for body weight, TP, and SC, respectively. Table-2 represents body weight and SC according to the presence of semen. The results showed that semen was collected when ram lambs presented 43.2±6.4 kg body weight and 25.8±3.7 cm of SC.

**Table-1:** Penile development stages (Stage 2: The penis is incompletely separated from the prepuce and Stage 3: The penis is completely separated from the prepuce) according to Tazegzawt ram lambs’ age, body weight, thoracic perimeter, and scrotal circumference.

Penile development stages	Age (weeks)	Body weight (kg)	Thoracic perimeter (cm)	Scrotal circumference (cm)
Stage 2	23.4±0.84	36.8±5.9	76.7±4.3	18.1±4.7
Stage 3	31.6±5.3	41.2±6.05	79±4.5	22.6±3.5

**Table-2:** Body weight and scrotal circumference in Tazegzawt ram lambs according to semen presence.

Body and testicular parameters	Semen absence	Sperm presence in the collected ejaculates
Body weight (kg)	36.9±5.8	43.2±6.4
Scrotal circumference (cm)	17.1±2.6	25.8±3.7

### Seminal parameters

**Figure-4:**
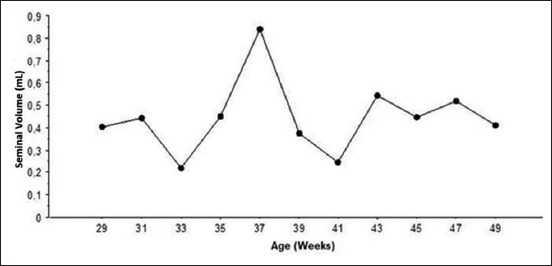
Biweekly seminal volume (mean±standard deviation) in Tazegzawt ram lambs from 29 to 49 weeks of age.

Electroejaculation was applied from 27 weeks of age, and the first semen samples were collected at 29 weeks of age from 30% of males. From then, semen was collected biweekly until 49 weeks of age; the percentage of the collected males increased gradually to reach 100% at 45 weeks of age. The mean semen volume during the study period was 0.48±0.32 mL with minimum (0.21±0.23 mL) and maximum volume (0.83±0.63 mL) observed at week 33 and 37, respectively (Figure-4).

**Figure-5:**
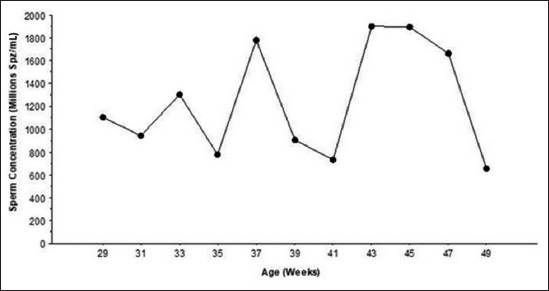
Biweekly sperm concentration (×10^6^) (mean±standard deviation) in Tazegzawt ram lambs from 29 to 49 weeks of age determined by the mean of Computer-Aided Sperm Analyzer.

Figure-5 shows that sperm concentration fluctuated considerably during the study period. When considering the total analyzed semen samples, a mean value was 1508±816×10^6^ spermatozoa/mL. The highest sperm concentration was observed at the age of 43 weeks with 1904±782×10^6^ spermatozoa/mL.

Gametes motility measured objectively using a computer analyzer showed that all kinematic parameters (Table-3) reached the highest values at the age of 49 weeks with 50.6±8.8%, 6.4±1.7%, 59.6±1 µm/s, 15.2±0.33 µm/s, 27.7±0.5 µm/s, 3±0.05 µm, and 4.2±0.08 hertz for motility percentage, progressive motility percentage, VCL, VAP, VSL, ALH, and BCF, respectively.

**Table-3:** Sperm kinematic parameters of Tazegzawt ram lambs obtained by the mean of Computer-Aided Sperm Analyzer (CASA) from 29 to 49 weeks of age.

Age	Motility (%)	Progressive motility (%)	VCL (μ/σ)	VAP (μ/σ)	VSL (μ/σ)	ALH (μ/σ)	BCF (Hertz)
Week 29	20.5±14.5	11.7±9.4	55.8±2.1	27.7±1.4	36.3±1.4	2.4±0.1	5.3±0.2
Week 31	18.3±9.8	8.8±6.0	50±1.6	28.3±1.4	35.7±1.4	2.1±0.1	5.2±0.2
Week 33	15.8±2.9	5.4±1.7	29±1.7	8.6±0.6	14.4±0.7	1.8±0.1	1.6±0.1
Week 35	24±5.8	6.7±2.4	39.7±1.4	28.3±0.5	62.9±0.7	7±0.1	4.4±0.2
Week 37	35.5±4.4	0.9±0.3	31.7±0.5	9.1±0.2	16.1±0.3	1.8±0.0	2.2±0.1
Week 39	25.3±2.2	0.1±0.1	22.8±0.6	4.5±0.2	9.7±0.3	1.5±0.0	1.4±0.1
Week 41	19.4±2.6	0.5±0.3	22.2±0.7	4.3±0.3	8.7±0.3	1.4±0.0	1.5±0.1
Week 43	25.4±3.6	0.3±0.1	25.7±0.3	7.3±0.2	13.8±0.2	1.7±0.0	1.5±0.0
Week 45	20.8±1.5	0.3±0.2	23.8±0.4	6.88±0.2	12.2±0.2	1.6±0.0	1.2±0.0
Week 47	31.5±10.3	4.4±2.8	45.7±0.5	14.6±0.2	22.9±0.3	2.4±0.0	5.0±0.1
Week 49	50.6±8.8	6.4±1.7	59.6±1.0	15.2±0.3	27.7±0.5	3±0.0	4.2±0.1

Data are presented as mean±SD. VCL=Curvilinear velocity, VAP=Average path velocity, VSL=Straight linear velocity; ALH=Amplitude of lateral movement of the head, BCF=Beat cross frequency, SD=Standard deviation

## Discussion

Tazegzawt sheep are well adapted to the local Algerian mountains; however, this breed remains not well studied and remains in an endangered status in relation to its small population. In our knowledge, no previous studies are available, especially concerning the reproduction physiology limiting substantially the initiation of preservation programs. The knowledge and mastery of reproduction particularly in males are one of the cornerstones in breeding management and preservation programs of endangered animals through semen biotechnologies.

Abraham *et al*. [13] reported that there are different definitions of puberty, including the presence of fully developed genitalia, libido, and a minimum concentration of sperm in ejaculate. Puberty in male is the moment when, for the first time, a male can mate a female and produce a pregnancy. Elhammali *et al*. [14] reported that the differences observed in terms of puberty onset are attributed to several factors including breed, climate, nutrition management, the methods of estimating puberty, and semen collection method employed.

In the current work, body weight, thoracic and SCs, penile development stages, and seminal parameters were considered to investigate puberty onset. Tazegzawt ram lamb presented a significant growth potential, at birth, lambs weighed 5.2±0.7 kg, and values were lower than those reported in Redkaraman lambs (5.6±0.33 kg [1]) but higher than those of several breeds including Bharat Merino (3.2±0.03 kg [15,16]), Romanov (2.5±0.18 kg [18]), Sardi (4.1 kg, [18]), D’Man (2.8±0.65 kg [16]), and Ouled Djellal (4.5±0.7 kg [19]), an Algerian breed which showed at one year age an average daily gain of 104.2±23.1 g/day.

Penile development stages revealed that semen was collected later after reaching the Stage 3. When reaching this stage, SC was 22.6±3.5 cm, values lower than those expressed at first semen collection (25.8±3.7 cm). This indicates that even if penile development is reported as an indicator of puberty onset, it remains not accurately related to spermatogenesis and semen production activity.

The results revealed that SC presented three growth kinetics: A slow growth from 9 to 21 weeks, exponential growth from 21 to 35 weeks, and a relatively stationary growth from 35 to 49 weeks of age. When using exclusively SC as an indicator of sexual maturity, puberty onset is considered as completed when SC presents the highest values. In fact, in the current results, puberty was observed, on the basis of semen collection, when SC was 25.8±3.7 cm (mean±SD), corresponding to the end of the exponential development stage.

In Tazegzawt sheep, males expressed puberty over 16 weeks (from 29 to 45 week) at 33±4.9 (mean±SD) weeks of age. Semen was first recovered from 30% of lambs at 29 weeks, and at 45 weeks, 100% of lambs reached puberty. Several authors indicate a quite considerable variation in age and body weight at puberty between and within breeds [20]. In fact, different ages at puberty are reported such as 228±7 days (31.5-32.5 weeks) in Ouled Djellal sheep [21], 27.3-28.7 weeks in Merinos sheep [9], 39 weeks in Yankasa sheep [22], 28.2±0.8 weeks in Santa Ines sheep [3], and 112.5±2.3 days (16 weeks) in Romanov sheep [18]. However, according to Chakraborty *et al*. [23], when using electroejaculation, the ram lamb could reach puberty 2 weeks earlier than what is recorded.

In Tazegzawt lambs, body weight, when compared to age, appeared as the most determinant factor conditioning puberty onset. In fact, from 29 weeks of age, collected males presented systematically higher body weight than the uncollected ones (43.1±5.5 kg vs. 35.3±8.9 kg); this seems to be in total accordance with the previous reports [11,22].

Body weight at puberty in Tazegzawt ram lambs (43.1±5.47 kg) appeared to be lower than those reported in Saidi ram lambs (50.3±3 kg [24]) but closer to those in Ouled Djellal ram lambs (40.4±1.2 kg [21]), Libyan fat-tailed ram lambs (39.9±9.3 kg [11]), and Suffolk sheep (36.8 kg; [20]). Lower body weights at puberty are also reported with 32.5 kg in Clun Forest sheep [7], 33.1 kg in Rahmani sheep [25], 24-29 kg in Merino sheep [9], and 23.7 ± 0.94 kg in Romanov sheep [18].

According to Ghorbankhani *et al*. [26], the semen produced by different breeds of sheep have been evaluated including essentially semen volume ranging from 0.6 to 1.6 mL and sperm density ranging from 2.6 to 5.5×10^9^ spermatozoa/mL. In Sanjabi ram lambs, these authors recorded 1.1±0.03 mL and 1112,3±67.7×10^6^ spermatozoa/mL for semen volume and sperm concentration, respectively. In Ouled Djella, an Algerian breed, Boussena *et al*. [21] recorded 1139±395×10^6^ spermatozoa/mL and 0.63±0.13 mL for sperm concentration and semen volume, values close to those of the current study.

## Conclusion

It can be concluded that the onset of puberty in Tazegzawt ram lambs born in spring, occurs between 29 and 45 weeks of age, body weight appeared as the most determinant factor.

## Authors’ Contributions

FM participated in the design and realization of the study, data collection, analysis, and interpretation and drafted the manuscript. RE participated in the design of the study. RN and NZ were involved in reviewing the manuscript. NA and MI designed and directed the research. All authors read and approved the final manuscript.
